# WebMaBoSS: A Web Interface for Simulating Boolean Models Stochastically

**DOI:** 10.3389/fmolb.2021.754444

**Published:** 2021-11-15

**Authors:** Vincent Noël, Marco Ruscone, Gautier Stoll, Eric Viara, Andrei Zinovyev, Emmanuel Barillot, Laurence Calzone

**Affiliations:** ^1^ Institut Curie, PSL Research University, Paris, France; ^2^ INSERM, U900, Paris, France; ^3^ MINES ParisTech, PSL Research University, CBIO-Centre for Computational Biology, Paris, France; ^4^ Equipe 11 labellisée Par la Ligue Nationale Contre le Cancer, Centre de Recherche des Cordeliers, INSERM U1138, Universite de Paris, Sorbonne Universite, Paris, France; ^5^ Sysra, Yerres, France

**Keywords:** Boolean modelling, stochastic simulation, web interface, SBML-qual, automatic mutations, sensitivity analysis

## Abstract

WebMaBoSS is an easy-to-use web interface for conversion, storage, simulation and analysis of Boolean models that allows to get insight from these models without any specific knowledge of modeling or coding. It relies on an existing software, MaBoSS, which simulates Boolean models using a stochastic approach: it applies continuous time Markov processes over the Boolean network. It was initially built to fill the gap between Boolean and continuous formalisms, i.e., providing semi-quantitative results using a simple representation with a minimum number of parameters to fit. The goal of WebMaBoSS is to simplify the use and the analysis of Boolean models coping with two main issues: 1) the simulation of Boolean models of intracellular processes with MaBoSS, or any modeling tool, may appear as non-intuitive for non-experts; 2) the simulation of already-published models available in current model databases (e.g., Cell Collective, BioModels) may require some extra steps to ensure compatibility with modeling tools such as MaBoSS. With WebMaBoSS, new models can be created or imported directly from existing databases. They can then be simulated, modified and stored in personal folders. Model simulations are performed easily, results visualized interactively, and figures can be exported in a preferred format. Extensive model analyses such as mutant screening or parameter sensitivity can also be performed. For all these tasks, results are stored and can be subsequently filtered to look for specific outputs. This web interface can be accessed at the address: https://maboss.curie.fr/webmaboss/ and deployed locally using docker. This application is open-source under LGPL license, and available at https://github.com/sysbio-curie/WebMaBoSS.

## 1 Introduction

The results of experimental observations often lead to non-intuitive behaviours, which could be explained, at the level of protein interactions, by unexpected cross-talks between two signaling pathways or by some gene alterations that could lead to unexpected phenotypes.

One way to cope with these issues is to formalize the knowledge about these pathways into networks. Some previous effort to standardize networks into formal representations (Systems Biology Graphical Notation, SBGN (Le Novere et al., 2009) has simplified their construction, use and analysis, and has also led to think carefully about the level of details and the types of information that is needed to answer a biological question. These networks are static and the analyses may be limited to fully address the problem from a dynamical perspective.

The translation of the biological processes into a mathematical model can shed some additional light on their functioning and can suggest possible scenarios to explain the experimental observations. Indeed, mathematical models simulating cellular dysfunctions have been studied for a long time to better characterize some cellular processes (Kholodenko, 2000; Tyson et al., 2002; Bauer et al., 2007), to recapitulate what is known about pathway deregulations or to predict possible points of intervention to revert some phenotypes associated to diseases (Lu et al., 2015).

There exist several mathematical formalisms that are best fit to answer a question depending on the data available, on the level of details required and on the types of results that are expected from the model. If the model is supposed to provide a precise drug dosage for a cancer treatment, partial or ordinary differential equations would be favored. These formalisms have successfully been applied to neuroblastoma (Fey et al., 2015) and melanoma patients (Gerosa et al., 2020). In this case, an important amount of information would be required to assign the proper values for parameters and initial conditions, which is not always possible.

If the model is built to suggest potential candidates for drug targets without quantitative details, a Boolean approach might be enough, which would not require a precise proper fitting of parameter values. Boolean networks have been successful in describing the regulatory and signaling networks of biological processes (Fauré et al., 2014; Flobak et al., 2015; Zañudo et al., 2019; Saez-Rodriguez et al., 2011). However, the types of conclusions or predictions that can be made with this approach are very qualitative.

To bridge these two formalisms, we have developed MaBoSS (Markovian Boolean Stochastic Simulator) which relies on a Boolean framework but uses a stochastic simulation approach by applying a Markov process on the Boolean network (Stoll et al., 2012, 2017). MaBoSS is a C++ software that exists in several formats: as a stand alone version that works on all platforms (MacOS, Linux and Windows), as a python library and as a web interface.

WebMaBoSS is a user-friendly tool that simulates Boolean models available in standard format using MaBoSS tool. What makes MaBoSS different from the other tools is that it associates parameter values to each variable of the model representing the speed of activation and inactivation of the corresponding node. With this framework, it is then possible to compute time trajectories over time, and provide probabilities for the model solutions (corresponding to a vector of the state of the variables). These probabilities can then be used to compare the behavior of the system in different conditions (e.g., wild type vs. mutated conditions). Any standard Boolean model, even if it was not meant to be simulated stochastically, can be used as an input and probabilities of reaching any state of the model can be computed.

To facilitate the use of our tool, we have already created a python library and Boolean model can be simulated through Jupyter notebooks in which MaBoSS functionalities can be embedded into a more in-depth analysis using other complementary tools from the logical community within the effort provided by CoLoMoTo (Levy et al., 2018; Naldi et al., 2018; Abate, 2020). What we wish to present here is an even simpler version, WebMaBoSS, which offers the possibility to study online, without any prior installation, any Boolean model, provided that they are available in standard formats. We provide an example of the web interface for which no details about the functioning of MaBoSS are needed. We show how to produce a simulation of the wild type conditions and of mutants (which can be understood as possible alterations of the wild type model), how to run some automatic mutations that can be assimilated to the search for the best drug treatment, and how to save the analyses and export images of the results.

## 2 WebMaBoSS: A User Friendly Tool to Simulate Regulatory Networks

### 2.1 Boolean Models

Boolean models are based on a coarse grain approach that considers that each variable of the model can take only two values, 0 or 1. A variable can represent a gene, a protein, or a metabolite. Since the pioneering works of Sugita (1963), Kauffman (1969) and Thomas (Thomas, 1973), Boolean networks have been more and more used to model regulatory and signaling network of biological processes such as development (González et al., 2008; Sánchez et al., 2002; Fauré et al., 2014), cell cycle dynamics in yeast cells (Fauré et al., 2006; Davidich and Bornholdt, 2008; Irons, 2009) or mammalian cells (Sizek et al., 2019; Traynard et al., 2016), and more specifically in the context of cancer (Saez-Rodriguez et al., 2011; Cohen et al., 2015; Zañudo et al., 2019; Checcoli et al., 2020). In some cases, multi-valued models, which consist of models with variables that can take more than two values, have been built to account for different phosphorylation states or for different gene or protein activities. These model can easily be translated into a Boolean model by duplicating the variables for each level and making the model purely Boolean, thus facilitating its computation and analysis (Didier et al., 2011).

Boolean models can be represented as a list of logical equations describing the update rules for each of the variables or/and as a wiring diagram, where nodes correspond to the variables of the model. In these networks, the causality between variables is depicted by a directed arrow illustrating a positive or a negative effect of one variable onto the others. Finally, the simulation of such models requires to choose an updating strategy, synchronous (where all nodes are updated simultaneously), asynchronous (where nodes are updated one at a time) or hybrid (combination of both synchronous and asynchronous) (Wang et al., 2012; Schwab et al., 2020; Garg et al., 2008), and can be applied to different types of Boolean models such as probabilistic (Shmulevich et al., 2002; Trairatphisan et al., 2013) or random Boolean networks (Harvey and Bossomaier, 1997).

### 2.2 Modeling Functionalities

There are numerous analyses that can be performed with a Boolean model. Among them, we can list: the study of model stability with the analysis of attractors including fixed points, limit cycles and their basins of attraction; the robustness of the model by modifying individual rules (interchange logical connectors within a rule) and estimating the changes in the model stability (comparison of attractors); a mutant analysis by studying the impact of knocking out or overexpressing some of the variables (which can be compared to a cell line or a patient mutational profile); further model perturbations can be done to simulate the impact on single and multiple drug treatments by automatically inhibiting nodes; contextualizing the Boolean model by integrating omics data; and many more.

If small or medium-size models are already very informative, simulating Boolean networks with a high number of nodes can be a complex task. Most methods rely on the construction of the state-transition graph, or some reduced version of it (Bérenguier et al., 2013). These methods do not scale well with an increasing size of the network, and make the simulation of models with a large number of nodes impractical, in particular with the asynchronous updating strategy. To cope with these issues, an alternative is to use simulations based on Markov chains, which consists in computing only the next possible steps, at each step of the simulation, and is by construction applying an asynchronous update strategy. In this case, the number of trajectories computed for each simulation needs to be high enough in order to ensure a proper coverage of the all state solution space, and a time long enough to reach the asymptotic solutions. The initial conditions can be set to a particular state or can be kept random to explore all possible behaviors. Moreover, even if all the nodes are used for the computation, only a selected set of output nodes are kept for the production of the results, thus giving a way to prevent the combinatorial state explosion.

With MaBoSS framework, the activation and inactivation of a Boolean variable can be separated and assigned a different transition rate, extending the algorithm to that of Gillespie (continuous time Markov chains), and that way, introducing the notion of physical time and the possibility to represent processes with different time scales.

### 2.3 Web Interface

We developed a web interface, WebMaBoSS, to target novices in modeling. Boolean models can be imported/exported in multiple formats (MaBoSS, SBML-qual, GINsim, BoolNet). Models are then stored in a database where they can be accessed at any time. They can also be directly imported from Cell Collective (Helikar et al., 2012) and BioModels databases (Le Novere et al., 2006).

WebMaBoSS can be accessed at the following address: https://maboss.curie.fr/webmaboss/. First time users can access the two pre-installed examples (Cohen et al., 2015; Corral-Jara et al., 2021) with a guest account and directly simulate them. For more advanced features, such as having their own private working directory, importing new models from files or databases, modifying models or performing sensitivity analysis, users need to register by providing a username and a password.

Once loaded, the network of a model, corresponding to the graph of interactions of the Boolean model, can be easily visualized via the web interface: the position of the nodes can be modified, exported as a png file, and the layout can be saved to be exported back into other software such as GINsim (by clicking on the disk icon in the Models working folder page).

The logical formulas of the model can be edited and checked for errors. The simulation parameters (initial values, output nodes, parameters and integration settings) can also be set and modified easily in the interface.

Once the simulation is executed, four types of results are plotted: time trajectories of both the model state probabilities and the node probabilities, a pie chart representing the probability of the last model states of the simulation, and a table of the fixed points computed and found during the simulation. Note that if the chosen maximal time is not long enough, the asymptotic solution may not have been reached and it is possible that not all stable states have been identified at the end of the simulation.

WebMaBoSS also allows the simulation of more complex tasks such as parameter sensitivity analyses. To run large sets of perturbations, the user can choose if he/she wants to test single or double mutants, with both activation or inhibition, from a list of selected nodes. This setting is particularly useful on big models, and should be chosen with care, because the number of double mutants can grow extremely fast. Once computed, the results of each mutant is printed, grouped by pages. The user can search for specific conditions or outputs and obtain the list of mutants producing the desired phenotypes.

Note that the number of outputs is limited to 15 to avoid very large computational load, and to prevent from having to store and to print on graphs very large amount of data. For the same reasons, the number of time points in a trajectory is limited to 100. Note that the number of outputs is limited to 15 to avoid very large computational load, and to prevent from having to store and to print on graphs very large amount of data. For the same reasons, the number of time points in a trajectory is limited to 100. Also, with MaBoSS, it is possible to define a complex initial state of model states (vector of nodes that may be linked in a particular biological setting) rather than of individual nodes as it is usually done. However, this cannot be set explicitly in WebMaBoSS but can be done externally in the configuration file (.cfg) directly and then reimported in WebMaBoSS.

### 2.4 Compatibility With File Formats and Databases

To ensure reproducibility of Boolean models and to facilitate the exchange between the available modeling software of the community, we ensured the compatibility with other common formats: BoolNet and SBML-Qual.

BoolNet uses a text-based format, allowing the possibility to write rules by hand in a simple manner (Müssel et al., 2010). SBML-Qual (Chaouiya et al., 2013) is a qualitative extension of the SBML core file format (Keating et al., 2020), the most common file format used in systems biology which focuses on quantitative modeling (ODEs). SBML-Qual can encode both Boolean and multi-valued models. To simulate models in MaBoSS, which only allows simulations of Boolean models, we implemented the method described by (Didier et al., 2011) to convert all models with multiple levels of activation into purely Boolean models.

The compatibility with these formats enable users to directly import models, through WebMaBoSS, from public databases of qualitative models such as Cell Collective (Helikar et al., 2012) or BioModels (Le Novere et al., 2006). As previously mentioned, an important added value of this functionality is the interoperability: users can easily build a model with software like GINsim (Gonzalez et al., 2006), Boolnet (Müssel et al., 2010) or CaSQ (Aghamiri et al., 2020), and simulate them using MaBoSS, inscribing MaBoSS and WebMaBoSS into a community combined effort to reproduce and exchange models.

### 2.5 MaBoSS Format, Particularities and Server Implementation

MaBoSS was initially built as a multi-platform software (Stoll et al., 2017) which can be run on command line. It uses two major files, one with the model description (bnd file), and one with the simulation settings (cfg file). The outputs are produced in a csv format. This usage, while very efficient, prevented a lot of potential users to explore MaBoSS. In 2018, python bindings for MaBoSS were developed and included in the CoLoMoTo jupyter notebook, which enabled to link MaBoSS to other modeling tools and to allow a new group of users to simulate already-built models with a stochastic approach (Levy et al., 2018). The CoLoMoTo environment also includes converter between formats, facilitating the import and export of models from different formats, although indirectly.

The analysis used with MaBoSS can require and produce a large number of simulations. The computational resources necessary to perform such simulation is thus critical. We developed a MaBoSS client/server implementation, where a server can be deployed to run MaBoSS simulations. MaBoSS client can connect to such server via a dedicated protocol, submit a simulation, and get the results. This allows to unload an important part of the computational resources on multiple, distributed servers. WebMaBoSS allows the configuration of such servers (host, port), which immediately become accessible to all users.

## 3 A Boolean Model Analysis With WebMaBoSS

WebMaBoSS can be accessed through a browser at https://maboss.curie.fr/webmaboss/. Registered users can organize and store the models inside a Project folder, where it is possible to upload a model in SBML-Qual or MaBoSS formats, or import a model from public databases such as BioModels and Cell Collective.

We illustrate the use of WebMaBoSS with an example of a published model of the regulation of the interleukins IL17 (Corral-Jara et al., 2021), which is accessible to guest users and already loaded into the default project of new accounts. Other model analyses and step-by-step tutorials can be found on the GitHub at the following address: https://github.com/sysbio-curie/WebMaBoSS.

### 3.1 IL-17A/IL-17F Differential Expression Model

To showcase a full study with WebMaBoSS, we studied a published model of T-helper cells for the regulation of IL-17A/IL-17F (Corral-Jara et al., 2021). The step-by-step tutorial showing all the functionalities of the web tool along with the interpretation of the results can be found in Supplementary File S1 and on the GitHub of WebMaBoSS (see above).

The model recapitulates the activation of the two cytokines IL-17A and IL-17F that are required for anti-microbial immunity and involved in auto-immune pathologies that can be linked to a subset of T-helper cells, Th1 and Th17.

The model encompasses different pathways organized in four modules: the TCR central module, that includes three different signals for the activation of naive T-cells, the Th7 module, that includes the cytokines leading to the Th17 phenotype, the IL-1*β* and IL-12 modules. The regulatory graph contains a total of 82 nodes and 136 interactions. The model analysis showed that NFAT2A, STAT5A and SMAD2 are key regulators of the differential expression of IL-17A and IL-17F.

As a support for the publication and with the goal to reproduce the results easily, several tasks, including the visualization of the stable states, the model analyses and the comparative studies were performed using the CoLoMoTo notebook which includes the python version of MaBoSS along with other modeling tools and can be found in the tutorial but also on the web repository of GINsim (http://ginsim.org/model/ThIL17diff).

**FIGURE 1:**
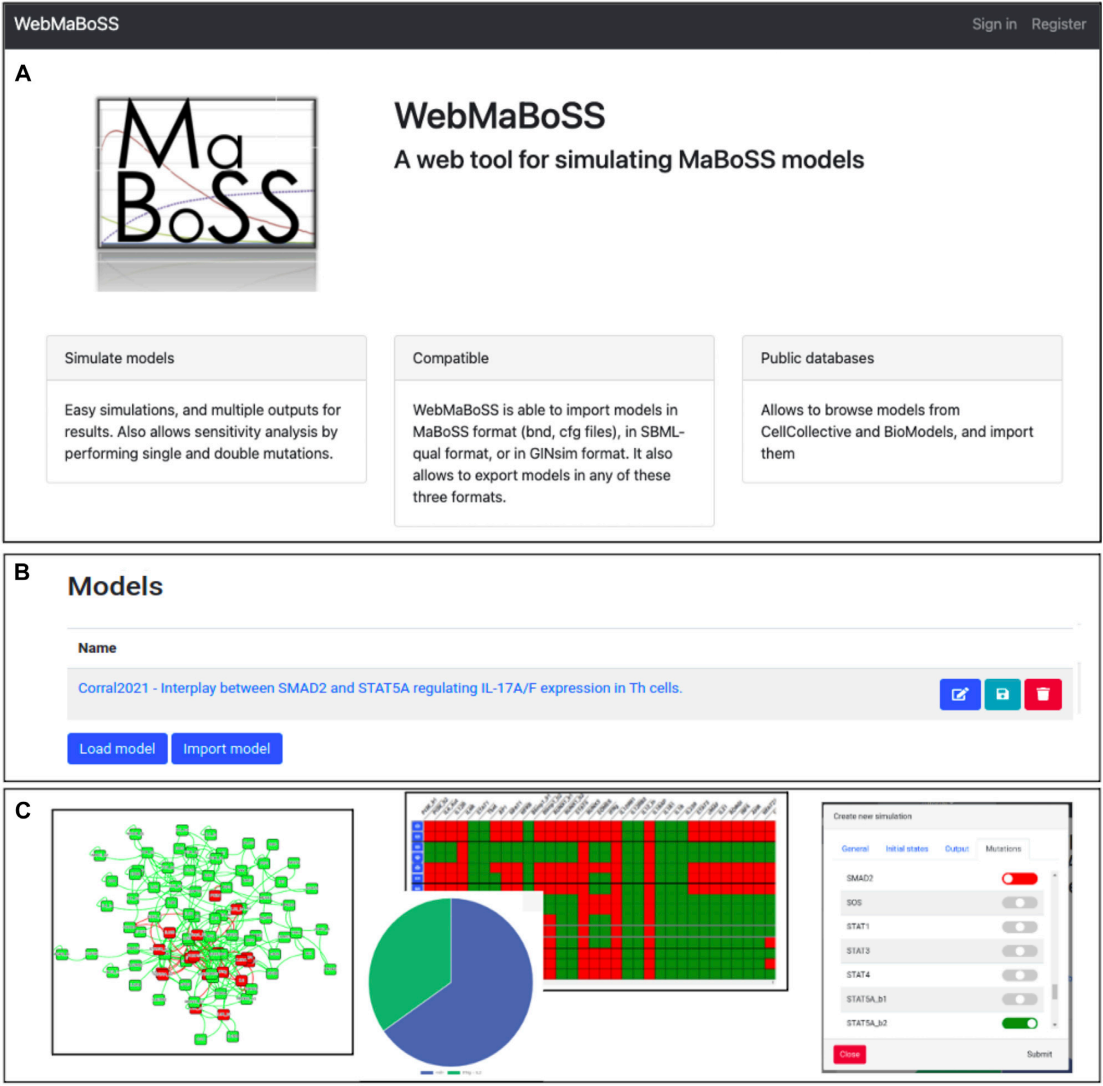
WebMaBoSS interface. **(A)** Web server welcome page of WebMaBoSS. **(B)** Loading or importing a model in WebMaBoSS. **(C)** visualization of the results: the network **(left panel)**, the simulation results with pie charts of the asymptotic solutions and the table of stable states **(middle panel)**, and the interface to perform more complex analyses such as mutant simulations or sensitivity analyses.

The model can be imported directly from the Cell Collective database by selecting the Import button (Figure 1B). The content of the file that contains the parameters (.cfg file) can be viewed in the Editing tab, where the user can modify the values of *Rates*, *Initial Values*, *Output*, *Parameters* and *Settings*. Note that the *Rates* tab can display the logical rules for each of the nodes of the model that can be modified directly in the interface, but new nodes can also be created.

The network of the model can be viewed in the interactive frame by clicking on the *Overview* tab: the initial layout is conserved, can be modified and imported back (Figure 1C, left panel). The results of the simulations can be visualized using several representations (Figure 1C, middle panel), e.g. as a table of fixed points, as a pie chart of the model states or as trajectories of either the probabilities of the model states or the node states. Some additional tasks can be performed such as mutant simulations by selecting, in the corresponding window, the nodes to alter (Figure 1C, right panel).

To reproduce the published results, we used three different initial conditions that correspond to three different configurations of input nodes presented in the initial publication, representing to the presence of a special combination of cytokines in the environment:

• Th1 condition, with initial state: IL12 (*IL12_In* in the model)
• Th17 condition with initial state: IL1 (IL1_In), IL23 (IL23_In), TGFB (TGFB_In), IL6 (IL6_In)
• IL-12 + IL-1b condition, with initial state: IL1 (*IL1_In*), IL12 (*IL12_In*)

**FIGURE 2:**
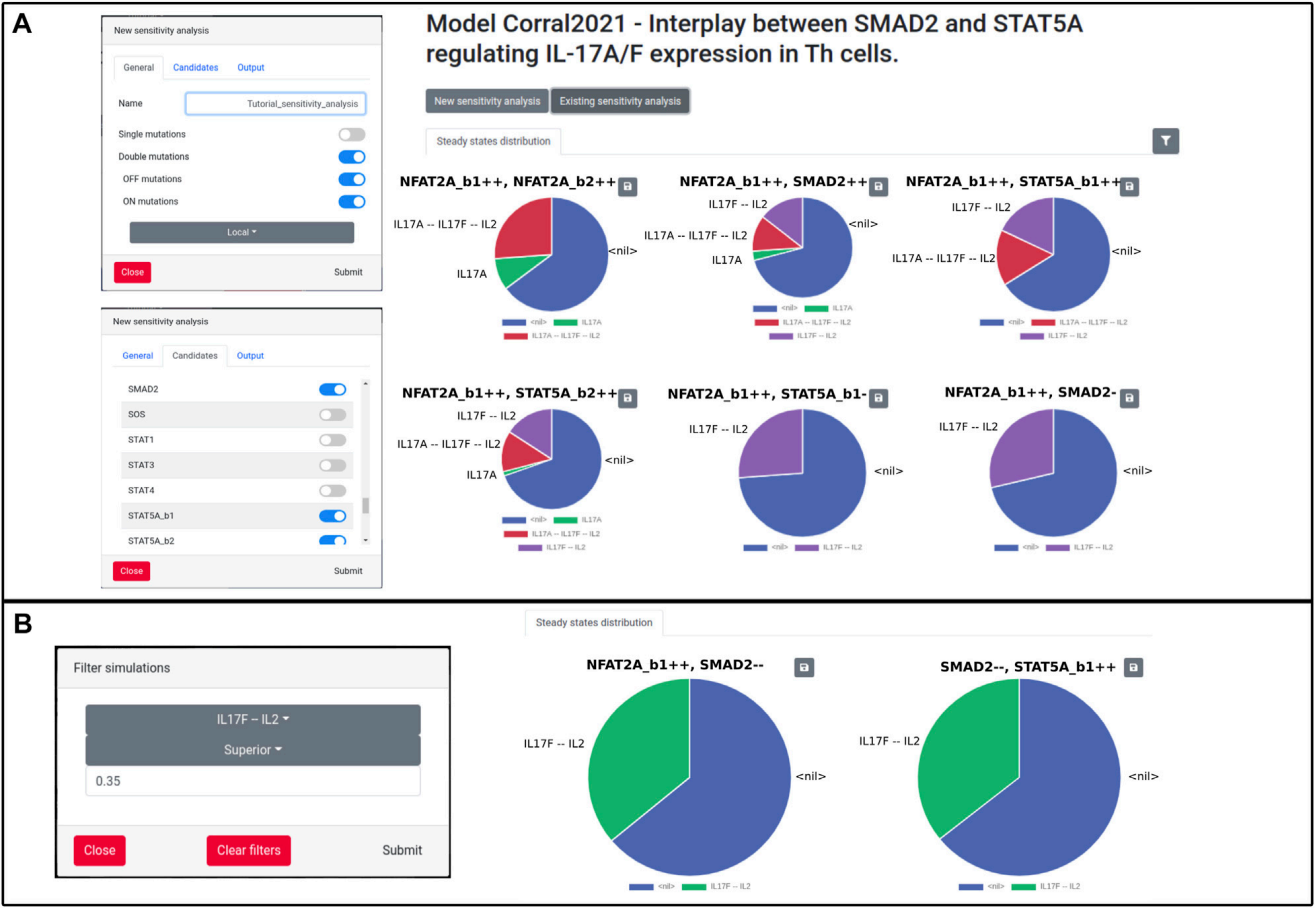
Sensitivity analysis on the Corral model. **(A)** Initial settings for simulations **(left panels)**, and visualization of the results of the simulations as pie charts **(right panel)**. **(B)** Filtering options’s window **(left panel)** and results of the filter for the IL17F–IL2 phenotype **(right panel)**

WebMaBoSS can also perform sensitivity analyses to check the effect of a knock-in/knock-out of each node on the network. The simulations will be launched one after the other, every time introducing a perturbation on a different node. This perturbation can be a single or a double mutation, representing a knock-out (OFF), an overexpression (ON) or a combination of both. To limit the length of the computation time, it is advised to select a subset of candidate nodes to explore the impact of the perturbations. WebMaBoSS then ouptuts the corresponding pie chart for each simulation and the user can filter the results to verify which mutation leads to the phenotype of interest. To demonstrate the importance of this functionality, we first simulate all the double mutants from a sublist of candidates (SMAD2, NFAT2A, STAT5A). We obtain a graphical list of all these mutants and their associated final states, which is in accordance with the published results (Figure 6 of Corral-Jara et al. (2021)). We can then filter the results and search for instance for mutations producing a large portion (more than 35%) of Th cells secreting IL17F, but not IL17A. For this example, we obtain two candidates for such a phenotype, with an inhibition of SMAD2 and an activation of either NFAT2A or STAT5A (Figure 2).

## 4 Conclusion

We present a web interface of the modeling software, MaBoSS, where Boolean models of various formats can be directly imported and simulated. This web tool offers the possibility to apply alterations to a model, in order to mimic mutations, disease conditions, etc. Automatic perturbations can also be performed, for parameters sensitivity analyses or model optimization for instance.

Therefore, Boolean formalism, within this web tool, provides a framework to test *in silico* hypotheses about the importance of some genes or pathways in cellular phenotypes, to suggest potential treatments with combinations of candidate drugs, etc. All these tasks do not require any prior knowledge of modeling or of the modeling tools. If biologists or non-modelers wish to play with a model, to try alterations that would mimic a disease or a patient profile, or to search for points of intervention to revert a phenotype (e.g., what to mutate to suppress the proliferative characteristic of a cell), they can easily test some ideas. We provide some tutorials in the GitHub repository and several examples on possible analyses and on the type of predictions that can be formulated from these simulations.

We are planning to extend some other functionalities of MaBoSS such as the integration of omics data to personalize the Boolean models of signaling pathways (Béal et al., 2019). The idea is to build individual models for patients in order to account for the data available [mutations, RNAseq, (phospho)proteomics data, etc.,] and modify the logical rules or the transition rates accordingly. A personalized model of a patient can already be simulated by hand by forcing the values of the nodes to the mutation profile of that patient. We plan to include this functionality to allow automatic construction of these models.

The purpose of the web tool is to simulate without any programming knowledge, however, for more complex simulations, we still advise to use the stand alone version or the python scripts. We provide Jupyter notebooks to facilitate its use in MaBoSS website (https://maboss.curie.fr).

## Data Availability

Publicly available datasets were analyzed in this study. This data can be found here: https://github.com/sysbio-curie/WebMaBoSS.
