# Supplementary material for: WebMaBoSS: A Web Interface for Simulating Boolean Models Stochastically
Source: Front Mol Biosci. 2021 Nov 15;8:754444. doi: 10.3389/fmolb.2021.754444 (PMC8651056; doi:10.3389/fmolb.2021.754444)

WebMaBoSS Tutorial

We showcase the different functionalities of WebMaBoSS with a published model [Corral et al., 2021]. Note that the notebook of the initial publication can be found here:

- ❖ Access WebMaBoSS through browser at https://maboss.curie.fr/webmaboss/.

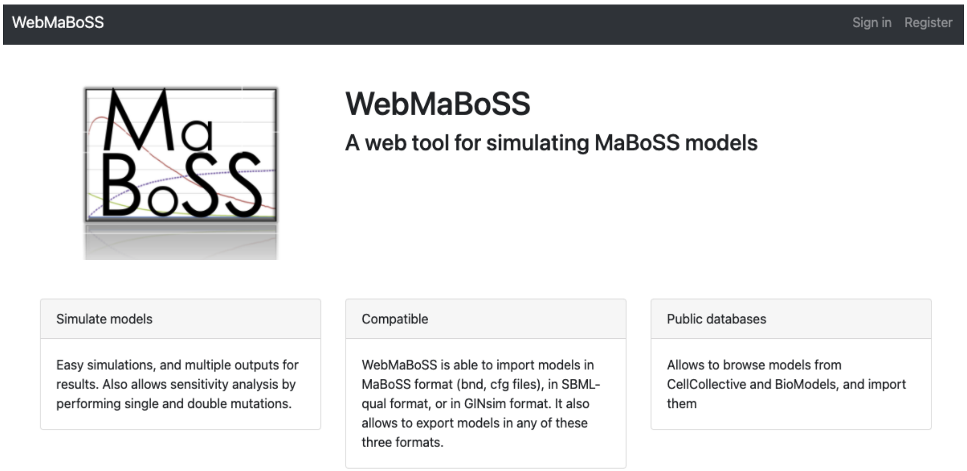

- ❖ Sign in / Register

If you access the server for the first time, you will have to register. Otherwise, enter your credentials.

- ❖ Starting a Project

WebMaBoSS allows the user to organize the models inside Projects folder.

You can create your own project by clicking on “New project”. You will associate a name and a description.

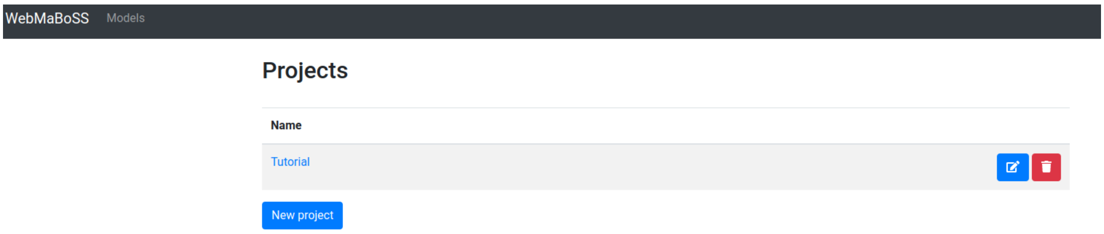

When you create a project, you have two options. You can:

- Load a model from your own desktop: in this case, you can choose between four predefined formats: MaBoSS, SBML-Qual, GINsim or BoolNet (BNet).
- Import a model from BioModels or Cell Collective: the server is linked to the content of the two databases and the models can be imported directly. In the future, we can extend

For this tutorial we study a model of T-helper cells for regulation of IL-17A/IL-17F from [Corral-Jara, K.F., Chauvin, C., Abou-Jaoudé, W. et al.] imported from BioModels.

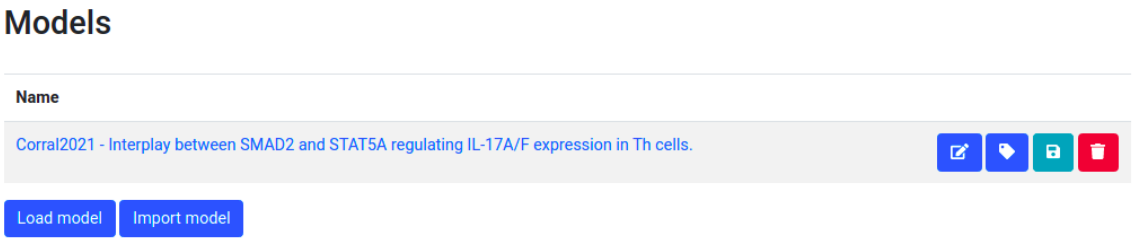

Once imported, the user can change the name of the model, export it in a new format or delete it. Clicking on the name of the model allows the user to edit and elaborate the model itself.

- **View the content of the model**
  - *Overview Tab*

Once imported, the model can be visualized. Note that the layout is conserved if the model is imported from GINsim where the layout is encoded in the model description.

Model Corral2021 - Interplay between SMAD2 and STAT5A regulating IL-17A/F expression in Th cells.

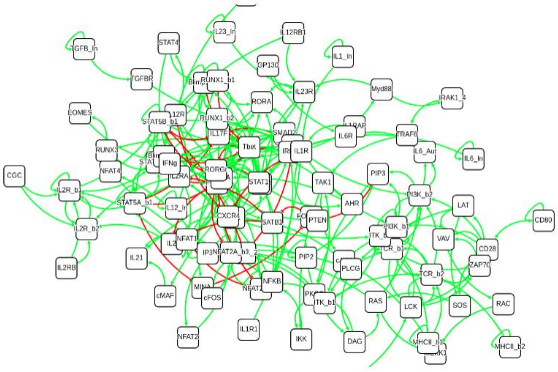

This model is composed of 82 nodes and 136 links, describing the interplay between different transcription factors involved in the regulation of IL-17A and Il-17F.

From this model analysis, it was concluded that NFAT2A, STAT5A and SMAD2 are key regulators of the differential expression of IL-17A and IL-17F.

- *Editing tab*

Models exported in MaBoSS are composed of two files with the extensions .bnd and .cfg: a *bnd* file for the model description (logical rules) and a *cfg* file for the setting of the model and simulation parameters.

With WebMaBoSS, the .cfg file can be accessed through the editing tab. The user can visualize the logical equations for each node. It is possible to: (1) edit the formula using the buttons on the right of the corresponding nodes or (2) insert a new node in the network by clicking on the "New node" button at the bottom of the list of nodes.

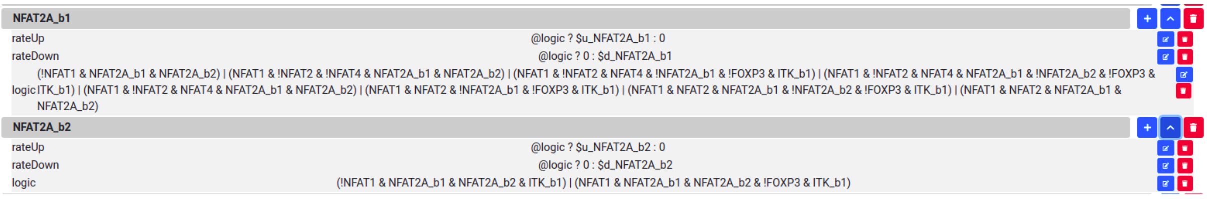

It is possible to set also modify the content of the .cfg file by setting different initial conditions, different outputs (variables shown explicitly in the simulations), transitions rates and simulation parameters.

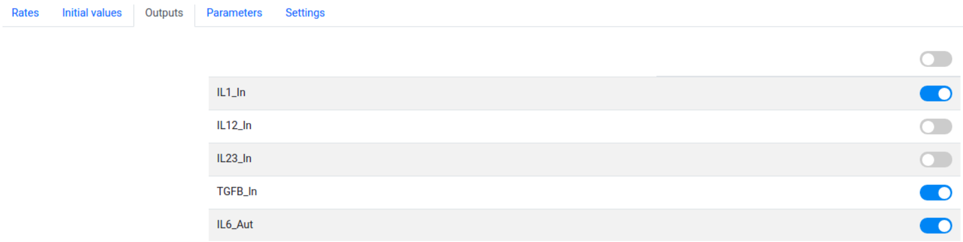

- *Simulation tab*

MaBoSS computes trajectories over time and the mean probability of the model states of interest. When launching a new simulation, one can name it, set the maximum time length, select the number of trajectories.

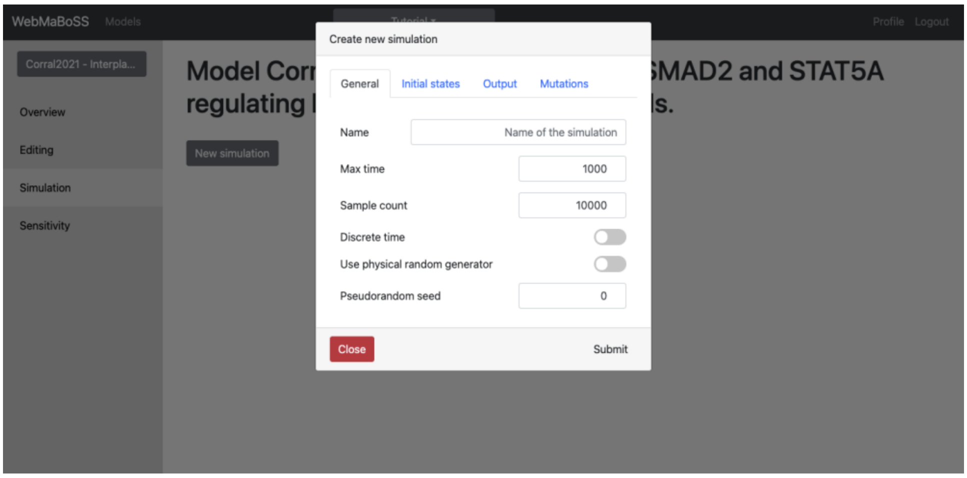

The initial conditions need to be set for each node. They can be all random or all set to 0 depending on the format of the files imported.

- Th1 condition, with initial state: IL12_In
- Th17 condition with initial state: IL1_In, IL23_In, TGFB_In, IL6_In
- IL-12 + IL-1β condition, with initial state: IL1_In, IL12_In

A subset of nodes is set to 1 (referred to as "common nodes"). On top of this definition of the "common" initial condition, these three configurations are set to account for different cell conditions. They can be found in the MaBoSS repository as .cfg file (see Corral_tutorial.cfg).

Another important point is to select the proper outputs for the simulations. MaBoSS uses all variables for computing the probabilities but if all variables are kept for the output, we may face a computational explosion. We suggest to choose only a subset of variables and launch several simulations with different outputs rather than keep all the variables of interest in the same simulation.

Below we are showing the results of the wild type simulations with different sets of initial conditions to replicate Figure 5 from Corral et al. For each figure, we used the same initial conditions presented in the notebook available in the supplementary material, in order to have a perfect match with the figure shown in the paper.

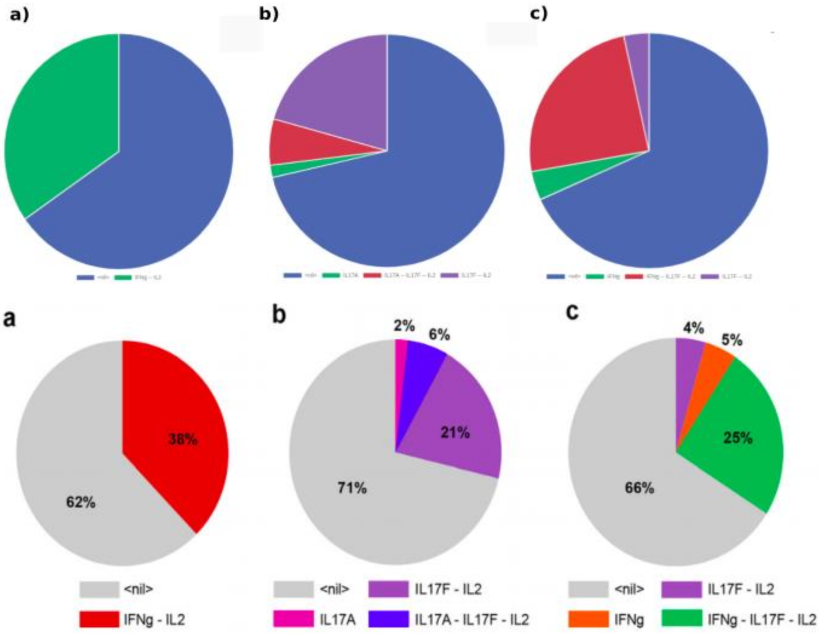

The results can be visualized as a pie chart (final state distribution), node probability trajectories, state probability trajectories, and fixed points.

- Nodes probability trajectories plot:

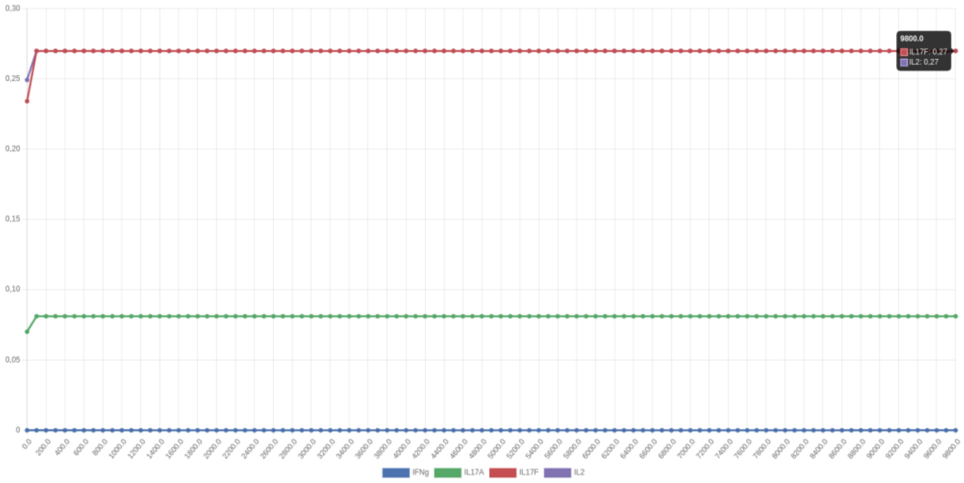

- Table of the **fixed points**:

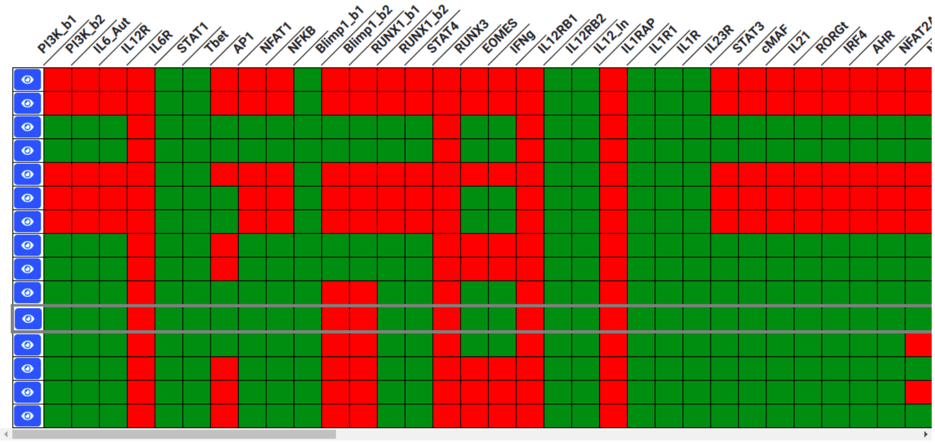

It is possible to visualize directly on the network the active nodes in the selected stable state, using the blue eye icon on the left.

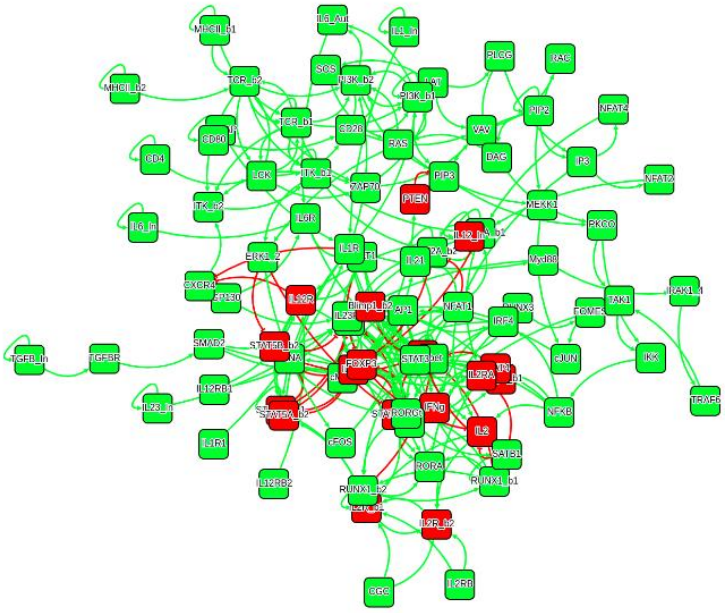

The Corral model proposes a combinatorial roles of SMAD2, NFAT2 and STAT5A in the differential expression of IL-17A and IL-17F. According to the table in fig. 6 of the initial publication, we can introduce a perturbation to check the different levels of expression of IL-17A based on the knock-in or knock-out of SMAD2 and STAT5A.

To introduce a perturbation in the model with WebMaboSS, the user can select one or more nodes in the Mutations tab and run a new simulation, as shown in the figure below:

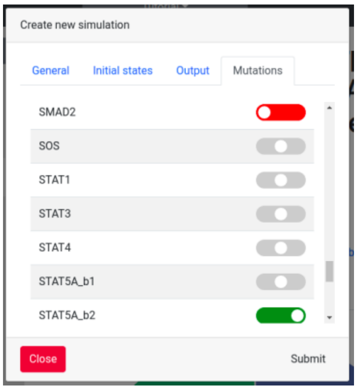

The perturbation can represent a gene overexpression (green switch) or a knock-out (red switch).

In the case shown above, we simulated with IL-12 + IL-1b condition, a knock-out of SMAD2 and a knock-in of STAT5A. This mutant leads to a higher activity of IL-17F and an inhibition of the activity of IL-17A, which, in the absence of SMAD2, is caused by STAT5A, itself activating IL-17F.

It is easy to verify the effect of the perturbation by looking at the States probability trajectories graph:

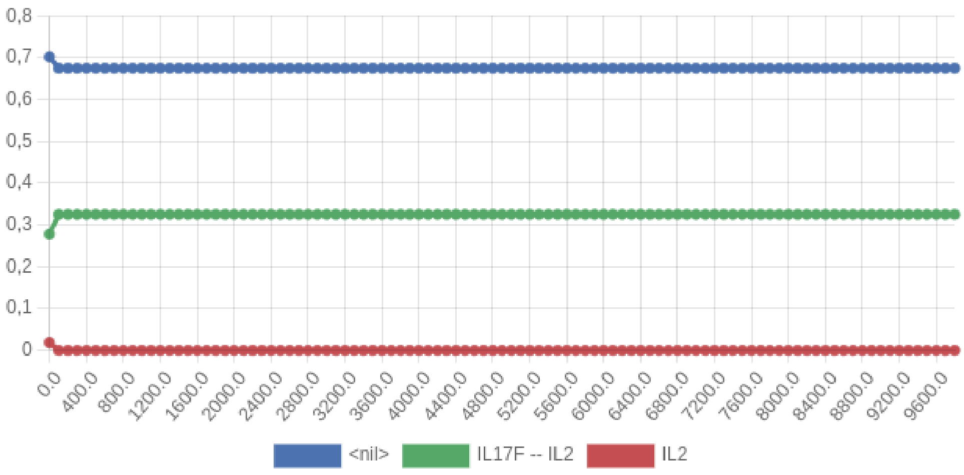

IL-17F is expressed along with IL2 while there is no expression of IL-17A.

- Sensitivity analysis

With WebMaBoSS, it is possible to perform a sensitivity analysis to check the effect of a knock-in/knock-out of each node on the network.

In the *General setting* tab, the user should decide whether to introduce a single or a double mutation and if this one would be a knockout (OFF), an overexpression (ON) or both. WebMaBoSS will simulate a perturbation on the model one node at a time (or two if the user select double mutation). Depending on the size of the network, this analysis will be quite expansive in terms of time, so, to avoid longer computational time, in the Candidates

tab, the user should select the interested nodes to mutate. Finally, as seen previously, in the Output tab, the user should select a subset of nodes to visualize to avoid a computational crash.

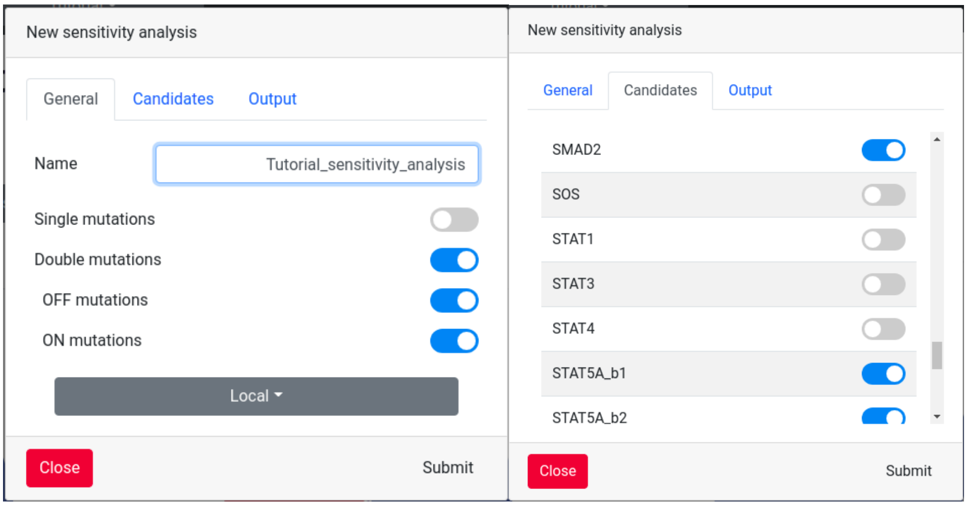

As example, we performed a sensitivity analysis of the model, choosing as candidates for the mutation SMAD2, STAT5A_b1, STAT5A_b2, NFAT2A_b1, NFAT2A_b2 and as output IL2, IL17A and IL17F.

At first, WebMaBoSS will print all the possible results, but using the filter function provided by WebMaBoSS, we can verify which mutation leads to a certain phenotype.

1) We want to look which mutations leads to a IL17A phenotype. We select the state we want to search for (IL17A) and a superior/inferior threshold (superior, 0.30). WebMaBoSS filters all the results until it finds the ones corresponding to the setting.

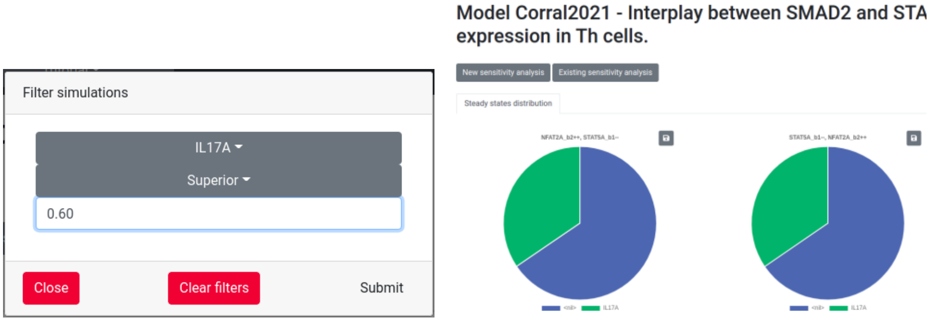

2) This time, we are searching for IL17F phenotype. We select in the filter the corresponding state (IL17F--IL2) and a threshold superior than 0.35:

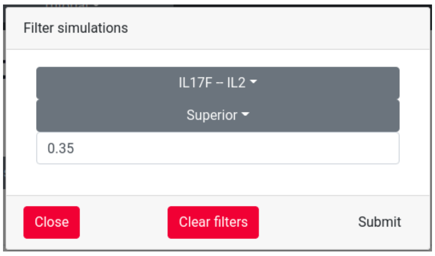

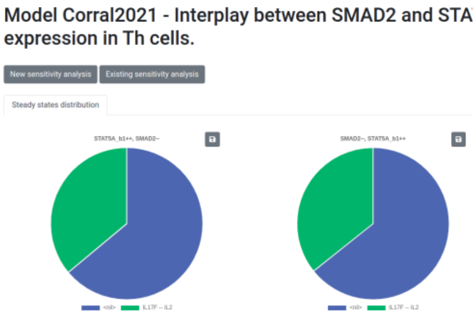

3) Finally, we search for both IL17F and IL17A phenotype, selecting the state IL17A—IL17F—IL2 with a superior threshold than 0.30:

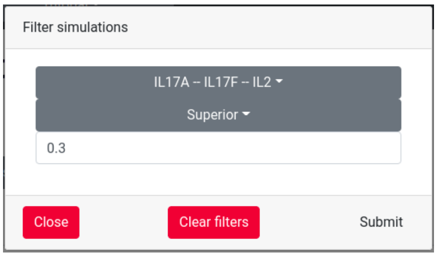

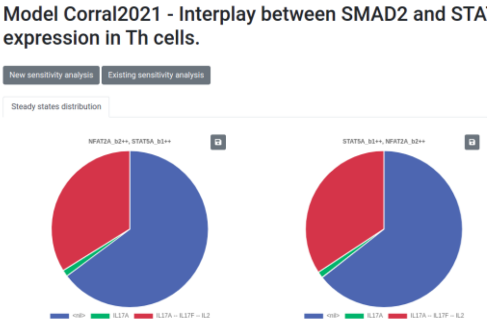

Supplement: Supplementary file 1 [file Presentation1.pdf]
